# Anatomy-Guided Microsurgical Resection of a Dominant Frontal Lobe Tumor Without Intraoperative Adjuncts: A Case Report from a Resource-Limited Context

**DOI:** 10.3390/diagnostics15182393

**Published:** 2025-09-19

**Authors:** Matei Șerban, Corneliu Toader, Răzvan-Adrian Covache-Busuioc

**Affiliations:** 1 Puls Med Association, 051885 Bucharest, Romania; mateiserban@innbn.com (M.Ș.); razvancovache@innbn.com (R.-A.C.-B.); 2 Department of Neurosurgery, “Carol Davila” University of Medicine and Pharmacy, 050474 Bucharest, Romania; 3 Department of Vascular Neurosurgery, National Institute of Neurology and Neurovascular Diseases, 077160 Bucharest, Romania

**Keywords:** neuroanatomy, clinical anatomy, glioblastoma, dominant frontal lobe, microsurgical anatomy, maximal safe resection, microsurgical technique, diagnostic imaging, resource-limited neurosurgery, functional preservation

## Abstract

**Background:** Glioblastoma (GBM), IDH-wildtype, is one of the most aggressive primary brain malignancies, and maximal safe resection is consistently recognized as a significant prognostic factor. Intraoperative adjuncts including functional mapping, neuronavigation, and fluorescence-guidance are not always present in many centers around the world. The aim is not to suggest equivalence to adjunct-assisted resections, but rather to illustrate the feasibility of anatomy-guided surgery in carefully selected cases and to contribute to the broader discussion on safe operative strategies in resource-limited environments. **Methods**: We present the case of a 54-year-old right-handed male who presented with progressive non-fluent aphasia, seizures, and signs of intracranial hypertension. Pre-operative MRI showed a heterogeneously hyperintense, frontobasal intra-axial mass involving the dominant inferior frontal gyrus, extending toward the corpus callosum and orbitofrontal cortex, and early subfalcine shift. Surgery was performed via a left frontobasal craniotomy, using subpial dissection and cortical–sulcal anatomical landmarks while aiming to preserve eloquent subcortical tracts (frontal aslant tract, superior longitudinal fasciculus). Nueronavigation, functional mapping or fluorescence was not used. We defined our outcomes by the extent of resection, functional preservation, and early radiological stability. **Results**: The procedure achieved a subtotal-near-total resection (>95% estimated volume) while maintaining functional motor function from prior to surgery and the patient’s baseline expressive aphasia, with no new neurological deficits. Early post-operative CT showed decompression of the resection cavity without hemorrhage or shift. At three months post-operative, CT showed stability of the cavity and resolution of the most perilesional edema with no evidence of recurrence. Clinically, the patient showed gradual improvement in verbal fluency, he remained seizure free, and maintained independence, which allowed for timeliness of the initiation of adjuvant chemoradiotherapy. **Conclusions**: We intend for the case to illustrate that, in selected dominant frontal GBM, following microsurgical anatomical principles closely may provide a high extent of resection with the preservation of function, even without advanced intraoperative adjuncts. We hope that our experience may support our colleagues who practice in resource-limited settings and contribute to our shared goal of both oncological outcomes and the quality of life of our patients.

## 1. Introduction

Glioblastoma (GBM), IDH-wildtype, is the most common and clinically malignant primary brain tumor in adults, representing around half of all malignant central nervous system tumors. According to the 2021 World Health Organization (WHO) classification of central nervous system tumors (5th edition), adult-type diffuse gliomas are now formally divided into three entities: astrocytoma, IDH-mutant; oligodendroglioma, IDH-mutant and 1p/19q-codeleted; and glioblastoma, IDH-wildtype [1]. This framework underscores the molecular definition of GBM, where the presence of TERT promoter mutation, EGFR amplification, or combined whole chromosome +7/−10 copy number change is sufficient to establish the diagnosis, even in the absence of classical histopathological features [2]. There is a well-established relationship between the extent of resection (EOR) and patient survival, with multiple prospective and retrospective series demonstrating consistently that if the EOR exceeds 90% cytoreduction then patients will survive longer. This must always be viewed in the context of potential irreversible (i.e., disabling) neurological deficits, especially for tumors of the dominant hemisphere where disabling aphasia, executive dysfunction, or motor deficits may have a major impact on quality of life [3].

The response of the modern neurosurgeon to this dilemma has been to adopt intraoperative adjunctive strategies such as neuronavigation, cortical and subcortical mapping, and 5-aminolevulinic acid (5-ALA) fluorescence. These strategies can improve surgical reliability, facilitate a greater EOR, and reduce functional morbidity [4]. However, access to these adjunctive surgical strategies is not uniform. In many centers, particularly in low and middle-income countries, the principal operative strategy remains as a microscope-based and anatomy-guided resection. Numerous audits of global neurosurgical practice have consistently reported on this disparity and have clearly demonstrated that the availability of access to intraoperative adjuncts differs geographically, economically, and systemically [5]. Not only does this gap exist within surgical practice, but it is also representative of the broader socioeconomic burden of GBM. Aside from high morbidity and mortality and years of life lost, treatment costs a significant amount of resources, creates considerable strain on caregivers, and vastly different amounts of healthcare are allocated in different regions with significant variability in outcomes [6]. The inequity in the availability of extended adjunctive technology is a direct example of how economic and infrastructural limitations influence day-to-day neurosurgical decision-making. In this environment, the surgeon is ultimately reliant on his or her substrate knowledge of the anatomy of the cortex and subcortex and the appropriate and refined intraoperative judgment to balance adequate oncological management and neurological protection [7].

With all this in mind, we report to you a large frontobasal GBM within the dominant hemisphere, managed by microscope-only, anatomy-based resection. This case demonstrates that reliance on pure anatomical landmarks is feasible and demonstrates some limitations of anatomy-only based resection when adjunctive strategies are not at hand. We aim to offer a pragmatic account of performing safe maximal resection in a low-resource setting.

## 2. Case Report

A 54-year-old right-handed male, with no previous history of neurological, epileptic, or neoplastic disease, presented to our neurosurgical clinic for evaluation of a progressive neurologic syndrome marked by the subacute emergence of expressive language deficits, seizure activity, and signs of intracranial hypertension. His past medical history included grade II essential hypertension, managed inconsistently over more than a decade, and associated grade II hypertensive retinopathy. He also had a background of cervical spondylodiscopathy (radiological stage IV) and multilevel lumbar degenerative disk disease, neither of which were symptomatic at the time of presentation. There was no family history of CNS tumors, epilepsy, neurocutaneous syndromes, or hereditary cancer predisposition.

Symptom onset occurred approximately two months before evaluation, with the gradual appearance of diffuse, dull, pressure-like bifrontal headaches. Initially infrequent and responsive to NSAIDs, the headaches evolved into daily episodes, pronounced in the early morning, often accompanied by nausea, non-bilious vomiting, and brief posture-related visual obscurations. The patient did not report photophobia, phonophobia, or fever. Over time, the headaches became refractory to over-the-counter analgesics, and his wife noted a subtle change in demeanor, with increasing fatigue, reduced social interaction, and diminished verbal output. The positional nature of symptoms, in conjunction with episodic visual symptoms, raised early concern for compensated intracranial hypertension, particularly in the context of his known hypertensive microangiopathy. Approximately three weeks prior to presentation, the patient began exhibiting clear signs of expressive language dysfunction. His speech became notably hesitant, with increased word-finding difficulty, simplified grammatical structure, and frequent pauses. Despite these expressive deficits, auditory comprehension and self-monitoring were preserved. The pattern was clinically consistent with a Broca-type non-fluent aphasia, suggesting involvement of the dominant posterior inferior frontal gyrus, possibly extending into the pars opercularis and pars triangularis, with suspected disruption of connected subcortical white matter tracts—including the frontal aslant tract (FAT) and anterior segment of the arcuate fasciculus. These pathways are known to subserve fluency, initiation of speech, and syntactic integrity, and may account for the progressive deterioration observed. Ten days prior to admission, the patient experienced a first unprovoked generalized tonic–clonic seizure, reportedly beginning with right perioral paresthesia and transient speech arrest, followed by loss of consciousness and bilateral tonic–clonic movements lasting under two minutes. He remained confused for several hours and was unable to speak meaningfully during the immediate postictal period. A second seizure occurred four days later, with similar semiology, including a focal onset in the right upper limb. No antiseizure therapy was initiated before clinic evaluation, and no prior EEG or neuroimaging had been performed. The clinical semiology strongly suggested a focal seizure of left frontal origin, most likely involving the dominant opercular-insular region, with secondary generalization. At the time of clinical assessment, the patient was afebrile, normoglycemic, and hemodynamically stable, though hypertensive (BP 162/98 mmHg). He was fully alert and oriented, with no signs of confusion or delirium. His mood and affect were appropriate, and there was no evidence of disinhibition, apathy, or psychomotor slowing. General physical examination was unremarkable. Dermatologic evaluation revealed no cutaneous lesions suggestive of neurofibromatosis or other phakomatoses.

Neurological examination confirmed a moderate to severe Broca-type aphasia, characterized by non-fluent, effortful speech, marked reduction in propositional language, impaired repetition, and word-finding pauses. Naming was delayed, and sentence construction was reduced to brief, telegraphic utterances. Comprehension remained intact for simple and moderately complex verbal instructions. Reading aloud was severely impaired, though silent reading was reportedly preserved. Writing was not assessed in detail at this stage. The language profile supported cortical involvement of the dominant inferior frontal gyrus, with possible extension into nearby frontal operculum and anterior insula. Cranial nerve examination was unremarkable. Visual fields were full to confrontation. Extraocular movements were intact, and no gaze deviation or nystagmus was observed. Pupils were equal and reactive to light. Fundoscopic examination revealed bilateral grade II papilledema, with blurred optic disk margins, venous congestion, and increased vascular tortuosity, but no hemorrhages or exudates—findings supportive of evolving intracranial hypertension. Motor examination revealed mild right-sided hemiparesis, with grade 4+/5 strength in both the right upper and lower extremities, spastic tone, brisk deep tendon reflexes, and a positive Babinski sign. A pronator drift was noted in the right upper limb. No cerebellar signs were elicited. Coordination was preserved bilaterally. Sensory examination was normal for all modalities, including fine touch, pinprick, vibration, and proprioception. Gait was not assessed due to recent seizures and the need to minimize fall risk.

Despite the expressive language impairment, the patient engaged effectively with non-verbal tasks. He demonstrated preserved attention, abstract reasoning, visuospatial organization, and pattern recognition, suggesting sparing of dorsolateral prefrontal, parietal, and temporoparietal integrative networks. His behavior remained appropriate, and insight was preserved. There was no evidence of mesial frontal, orbitofrontal, or limbic disinhibition. Formal neuropsychological testing—including fluency tasks, semantic categorization, and symbolic sequencing—was planned but deferred until stabilization, given the need for urgent neuroimaging and seizure control. The patient’s systemic profile, notably his chronic hypertension and established microvascular disease, warranted consideration of small-vessel ischemic comorbidity, which could contribute to perilesional white matter changes or seizure threshold reduction. However, the pattern of clinical evolution—including progressive non-fluent aphasia, localizing seizures, right corticospinal signs, and objective papilledema—was most consistent with a space-occupying lesion in the dominant frontobasal region, exerting mass effect, producing cortical and subcortical dysfunction, and likely causing subfalcine shift. A rapidly expanding high-grade glial neoplasm, such as a GBM (IDH-wildtype), was considered the leading diagnostic possibility, based on age, semiology, and time course. Differential diagnoses included anaplastic astrocytoma, oligodendroglioma with secondary transformation, and —though less likely—primary CNS lymphoma or tumefactive demyelinating disease. Basic laboratory workup—including CBC, serum electrolytes, and renal and hepatic panels—was within normal limits, and there were no signs of systemic inflammation, metabolic instability, or infection.

**Figure 1:**
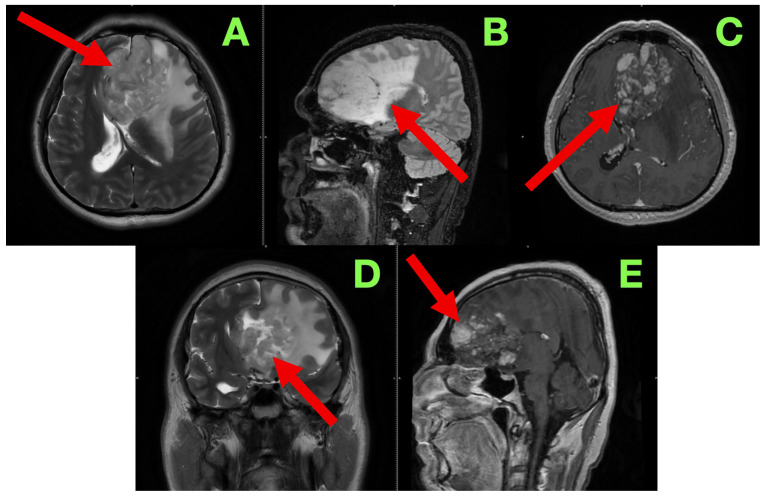
Pre-operative magnetic resonance imaging of the brain. (**A**): Axial T2-weighted image showing a heterogeneously hyperintense, poorly marginated intra-axial lesion (red arrow) centered in the left inferior frontal gyrus, with extension into the middle frontal gyrus and deep white matter. Marked vasogenic edema effaces cortical sulci, compresses the left lateral ventricle, and produces a midline shift of approximately 7 mm. (**B**): Sagittal FLAIR sequence demonstrating tumor extension into the subcallosal area (red arrow), with superior displacement and anterior bowing of the genu of the corpus callosum. Narrowing of the interhemispheric fissure and blurring of adjacent gyri indicate early subfalcine herniation. (**C**): Axial post-contrast T1-weighted image revealing patchy, nodular enhancement (red arrow) at the superomedial margin of the lesion, without central necrosis or ring formation. This enhancement pattern suggests partial disruption of the blood–brain barrier, typical of an infiltrative glial process. (**D**): Coronal T2-weighted image demonstrating inferior tumor extension into the frontoorbital cortex and anterior insula (red arrow). Associated asymmetric edema exerts mass effect on the anterior cerebral artery territory. The left frontal horn is compressed, and medial structures are displaced. (**E**): Sagittal post-contrast T1-weighted image showing faint, non-uniform gyriform enhancement (red arrow) along the frontobasal convexity, with blurring of the cortical–subcortical junction. No infratentorial involvement or leptomeningeal enhancement is present.

The patient was admitted for further diagnostic workup, seizure prophylaxis, and early surgical planning. Contrast-enhanced magnetic resonance imaging (MRI) (Figure 1) was immediately scheduled to characterize the lesion’s size, location, vascular behavior, potential infiltration of eloquent structures, and operability.

In order to better characterize the lesion and its relationship to eloquent brain structures, we performed a contrast-enhanced cranial MRI following commonly accepted practices and standard high-resolution protocol, which included T1-weighted, T2-weighted, FLAIR, and post-contrast T1 sequences, acquired in the axial, sagittal, and coronal planes. Our goal was to evaluate not only the lesion in terms of size and morphology, but patterns of infiltrative growth, behavior of contrast, edema in the perilesional area, and any early signs of herniation or distortion of local tissue—all useful information in formulating a surgical approach. The study showed a large intra-axial lesion, centrally located in the left inferior frontal lobe, extending medially across the interhemispheric fissure, and abutting potentially or involving structures including the genu of the corpus callosum, anterior cingulate gyrus, and the subcallosal area. The heterogeneity of the lesion suggested infiltrative or infiltrating capacity, with indistinct margins and associated mass effect on surrounding gyri and white matter tracts. In the axial sequences on T2-weighted images (Figure 1A), the lesion exhibited heterogeneous hyperintensity, made up of heterogeneous, irregular, infiltrative borders. The lesion extended from the inferior frontal gyrus into the middle frontal gyrus (pars triangularis and opercularis), with evidence of perilesional signal change indicating vasogenic edema, extending inferiorly from the deep portions of the frontal lobe into the critical centro-semiovale, frontal horn, and anterior portions of the corona radiata. The left frontal horn of the lateral ventricle was compressed, and a midline shift of approximately 7 mm was observed. There was also some subtle effacement of the left Sylvian fissure and perisylvian cortex evident. The overall findings suggested not only cortical but also MCU of some important white matter tracts, particularly the FAT and pairing superior longitudinal fasciculus (SLF), and potentially less so of the anterior limb of the internal capsule. The sagittal FLAIR images offered further insight regarding vertical and anteroposterior extent of the lesion. There appeared to be deformation of the genu of the corpus callosum as it looked bowed posteriorly and superiorly. Edema extended anteriorly toward the cingulate gyrus and there was narrowing of the interhemispheric fissure. The lesion was clearly in contact with the subcallosal area and there appeared borderline indistinctness from the gyrus rectus and the olfactory sulcus as well as possible involvement of ventromedial prefrontal regions. The basal cisterns were patent but due to evident asymmetries in midline structures, likely early subfalcine shift was occurring. Axial post-contrast T1-weighted images (Figure 1C) showed multifocal nodular enhancement but without any significant or well-formed ring enhancement or associated central necrosis. Areas of contrast uptake were seen at the superior and medial margins of the lesion and there was an area extending towards the callosal–cingulate junction. These findings could represent frank blood–brain barrier disruption and absent necrotic transformational features. There was importantly no enhancement of the ependyma or ventricular dissemination or leptomeningeal spread. Coronal T2-weighted images (Figure 1D) confirmed inferior extent of the lesion and effacement towards the orbitofrontal cortex and adjacent anterior insula. There was good correlation of sulcal effacement to these areas. The finding appeared to laterally displace the medial frontobasal cisterns, and surrounding edema was located toward the limen insulae. These findings raised the question of possible subpial invasion with respect to language relevant structures in the perisylvian area. Beyond the limitations of the axials, the sagittal post-contrast T1 (Figure 1E) images also demonstrated blurring between the cortical–subcortical transition in the convexity of the frontoorbital region and focal enhancement along the gyral surfaces, perhaps indicative of either superficial cortical spread or diffuse perivascular pathology. Spatially, the observed enhancement was patchy and irregular—reasonably suggestive of an infiltrative neoplastic process, but not specific. There were no signs of enhancement of the basal meningeal layers or of infratentorial structures. The third and fourth ventricles were symmetric and non-dilated.

Read in conjunction with the clinical presentation, the radiologic findings shaped our impression of an infiltrative high-grade glioma—most likely a GBM (IDH-wildtype). Out of an abundance of caution with diagnosis, we also considered the option of an anaplastic astrocytoma, as well as the potential for secondary transformation of a lower-grade glioma, and, with the least likelihood, primary CNS lymphomas or tumefactive demyelinating lesions, given the absence of necrosis and ring enhancement. The degree of mass effect and the nature of the enhancement noted seemed excessive relative to contrast uptake, which we reasoned as representing a high-grade glioma likely to have diffuse infiltrative behavior with minimal central breakdown.

The involvement of the dominant inferior frontal gyrus, in particular, with adjacent extension in and around regions absolutely necessary for speech initiation and fluency, aligns with the patient’s presentation of non-fluent aphasia. Also, even the surrounding edema encroaches toward the anterior limb of the internal capsule. This correlates with the positive right-sided corticospinal findings on examination. Additionally, we were concerned about the radiologic appearance suggesting ‘early’ subfalcine herniation. That said, at this phase, there was no evidence of distortion of the brainstem structures or uncal shift.

To prepare for surgery, we hoped to coordinate these aspects into a prognosis that yielded a functional-expanding approach. We suspected it would be difficult due to the vascularity of the lesion, the infiltrative margin, and the proximity to language cortex and the related tracts. Surgical resection was performed with the goal of achieving maximal safe debulking and was entirely reliant on knowledge of anatomy and intraoperative observation. In light of the infiltrative character of the lesion, the frontobasal depth of the lesion, and the patient’s dominant hemisphere, a traditional microsurgical approach was selected. We intentionally performed the procedure without neuronavigation, intraoperative mapping, and 5-ALA fluorescence techniques. This was with the intent of maintaining our attention on tactile and visual motors of the operation and respecting the anatomy revealed in real time, an approach honed through years of experience treating lesions in this region of the brain that often present with subtle topographic nuances. The patient was placed in a supine position with the head turned about 25–30 degrees right relative to the table and held in a three-pin cranial fixation device. The head was modestly extended to make the left frontal convexity orthogonal to the surgical field. This also satisfied our goal of using the retraction of gravity to expose the basal frontal structures with minimal displace. Our efforts were to create a working corridor to the inferior frontal gyrus, frontal pole, and subcallosal area without anteriorly deviating the hemisphere to put non-desired tension in the bridging veins and possibly separating the basal cistern structures. A left frontotemporal curvilinear scalp incision was made and a single-piece frontobasal craniotomy was raised with three burr holes (keyhole, frontal squama, and pterion) located to facilitate maximum bone removal inferiorly. The extent of the craniotomy enabled exposure from the frontal pole to the Sylvian fissure, sustained exposure to the inferior frontal sulcus, and allowed subsequent access to the adjacent opercular cortex. During elevation of the frontobasal craniotomy, the frontal sinus was carefully avoided, and no entry into the sinus cavity occurred. This minimized the risk of post-operative CSF leak or infection, which can complicate anterior cranial fossa approaches. With exposure to the cranial cavity, the dura was observed to be tense and no pulsatility could be observed, validating our interpretations from the modality imaging of suspected intracranial pressures issues. The dura mater was opened in a C-shaped anteriorly based flap, which was reflected anterolaterally and fixed to the bone lip. The brain just below it was globally edematous with pale, flattened gyri and poor sulcal definition. There was no visible exophytic component or surface discoloration, as expected for deeply seated, infiltrative lesions. To decrease cortical tension and avoid excessive retraction, the left opto-carotid cistern was opened to allow controlled CSF drainage, with direct and careful vision and microdissection of the fine arachnoid, respecting the optic nerve sheath and anterior perforators. The operating microscope was positioned and microsurgical dissection commenced. A subpial entry point was selected on the posterior segment of the left inferior frontal gyrus, carefully 1.5–2 cm anterior to the presumed precentral sulcus, lateral to the midline, and posterior to the frontal pole—a trajectory that balanced reasonably safe access and preserving motor and supplementary speech regions.

Corticotomy was performed along the inferior frontal sulcus using a fine bipolar tip and microsuction. Upon accessing the tumor tissue, it was gray-white, friable and somewhat vascular, and without any discernible capsule or margin. The consistency was soft, and aspiration brought bits of granular tissue and some microvessel ooze, suggesting a possible high grade astrocytic lesion. There was no plane of cleavage between tumor and brain, and so excision was performed carefully, developing a centripetal envelope in a piecemeal fashion, using alternating bipolar coagulation, fine suction, and blunt microdissectors.

As resection continued, the temptation to evaluate how much tumor was being resected was constantly modified by dependence upon visual, tactile, and anatomical reflexes rather than technology. Tumor tissue was described as soft, gray-white tissue, as opposed to elastic, organized tissue of spared parenchyma, and this was the first indication of operative limits. When the distinction of tumor and parenchyma became unclear and the plane of cleavage between infiltrated tissue and inflamed but functioning cortex was lost, resection would inevitably slow, and ultimately cease, as the risk of resecting eloquent tissue was now greater than any potential oncological benefit. The resection progressed in the surgical field, and the texture of the tissue and organization of spared medullary fibers provided other indications. For example, increasing resistance and firmness indicated that one had most likely entered tracts that were eloquent, especially along the posterior margin where the appearance of the anatomy located the anterior limb of the internal capsule. At this point in the operation, the method went from aspiration to preservation, where even a slight transgression could translate to a permanent and profound deficit.

Vascular structures also provided important reference points for surgical judgment and decision-making. In addition, the existence of small perforators from the anterior cerebral artery along the cingulate sulcus and small vessels off the recurrent artery of Heubner—along with veins returning to the anterior perforated substance—created anatomical demarcation lines indicating limits that can be approached but not crossed. Each of the vessels was preserved by careful sharp dissection and low energy bipolar coagulation, and the presence of the vessels represented that we had stopped resections in those planes. The anatomical borders delineated by the risks in the midline and subcallosal area were equally self-evident. At this point, the dissection was close to the genu of the corpus callosum and area of the subcallosum and was moving into the risk of crossing fornical columns or crossing ependymal surfaces. It was important to respect these deep structures because their injury would compromise memory and executive function and overall quality of life. The risk of uncontrolled bleeding was also an important limiting factor. In the areas that would have required a lot of coagulation where very delicate perforators or venous collectors were in the way to carry out the next step to aspirate, we simply left it alone. The unilateral decision was made much easier in the absence of intraoperative mapping: without awake speech and language testing, any depth of dissection that was concerning on some level for resistance, fiber orientation, and distortion of anatomy as it related to involvement of the frontal aslant tract and superior longitudinal fasciculus constituted a functional red line and was treated as such. Under the contingencies provided in this section, the decision-making evolved through intraoperative evaluations made by composite tactile sensations, visual observations, and vascular–anatomical cautions in a process that allowed the resection to proceed until all margins were determined—not on the basis of technical ability, but rather on risk related to function or anatomy.

The freedom to respect the superficial and lateral aspects of tumor mass to a level that produced decompression and a normalization of contour was possible, while the deep frontobasal and medial extensions of tumor mass were acknowledged and intentionally left in place. While the cavity itself looked grossly clean in the fields we could visualize, we made the explicit decision to respect the anatomical limitations and end up with a subtotal rather than a gross total resection. Thus, the disease that was left was not a technical limitation, but a conscious decision to respect a surgical limitation, to protect the eloquent cortex as well as vascularity, and facilitate the patient to proceed to adjuvant therapy without additional risk.

Medial advancement provided access to the pericallosal area, with the genu of the corpus callosum clearly angled posteriorly, which resulted from medial bulk from the tumor, likely. We intentionally stayed within callosal fibers and cingulate gyrus, stopping just short of direct transcallosal violation. The dissection in this corridor was particularly interesting in that it required very sharp definition of planes since many vascular landmarks were obscured by edema and gliotic reaction. Along the cingulate sulcus, we encountered three small pial perforating vessels from the anterior cerebral artery that we preserved through very sharp dissection and low energy bipolar. Inferiorly, the lesion tapped into the gyrus rectus and medial orbitofrontal cortex, with swelling almost differentiating the olfactory sulcus. We preserved the olfactory tract, noting compression, but not displacement. Special care was taken to ensure its preservation, and post-operatively the patient did not report subjective anosmia, with olfactory function appearing clinically preserved. As we continued deeper with dissection, we paid particular attention to our dissection planes with respect to the subcallosal area, where the anatomical realizations of margins would almost challenge any use of a microscope. We did not directly visualize the fornical columns but were estimating through depth and trajectory; while cognizant of our functional restraint throughout our depth, we also respected midline ependymal structures at all costs. As we dissected laterally, we were approaching the anterior insular border and did not formally enter the Sylvian fissure. At the posterior extent, the tumor was at what appeared to be an anterior limb of the internal capsule, based on tissue tensile firmness, but also the orientation of any surviving medullary fibers and the deep perforating vessels coming off a Heubner artery and anterior perforated substance. This part we treated as a silent surgical margin, where any resistance at depth and perception of risk for bleeding was interpreted as functional stop point—which again we chose to respect, stopping all surgical activity at both the deep and medial tumor extents.

We were able to safely grossly resect the more superficial and lateral portion of the tumor; however, due to vascularity, the anatomic distortion of surrounding structures, and their proximity to eloquent subcortical tracts, we intentionally left small volumes of the deep-seated frontobasal lesions intact. Although the resection cavity appeared macroscopically clean in its superficial and lateral aspects, small volumes of deep frontobasal tumor were intentionally preserved. Based on intraoperative judgment and post-operative imaging, the overall extent of resection was estimated at approximately 90–92%, corresponding to a subtotal resection. It is well recognized that earlier surgical intervention, performed before severe edema or neurological deterioration, generally facilitates safer cytoreduction and smoother post-operative recovery, whereas delayed surgery in the presence of worsening deficits or raised intracranial pressure may increase the risk of morbidity. In this case, surgery was timed promptly after diagnosis, enabling maximal safe debulking while preserving function.

A hemostatic environment was created through staged bipolar coagulation, and as required also using oxidized cellulose (Surgicel) and hemostatic matrix. After irrigating the cavity with warm Ringer’s lactate and observing no breach of an ependymal barrier or compromise of venous access or return, we conducted a final microscopic inspection and noted a clean, satisfactory hemostatic bed without any residual exophytic component. The dura was closed in a primary fashion using interrupted nonabsorbable sutures, followed by a replacement of the bone flap and layered closure. A subgaleal closed suction drain was placed. There were no complications at surgery.

The patient was transferred, intubated and hemodynamically stable, from the operating theater to the neurosurgical intensive care unit for vigilant monitoring in the immediate post-operative period. He was sedated for one hour after arrival and clinically extubated without incident, exhibiting spontaneous respiratory effort, persistent full consciousness, and a Glasgow Coma Scale score of 15. The patient was alert and oriented to person, place, and time, and able to follow a series of complex commands. The initial neurological examination yielded no new motor deficits. There was no drift, with the right hemibody strength (5/5) preserved, and no focal paresis. The cranial nerve examination was unremarkable. The patient’s language output remained hypophonic and slow, and reflective of the baseline level of expressive aphasia, with no further deterioration. There was no evidence of dysphasia, ideomotor apraxia, neglect, or visual field deficit. Pupils were equal and reactive, and there was no evidence of papilledema on fundoscopic examination. The surgical site dressing was clean, dry, and intact.

**Figure 2:**
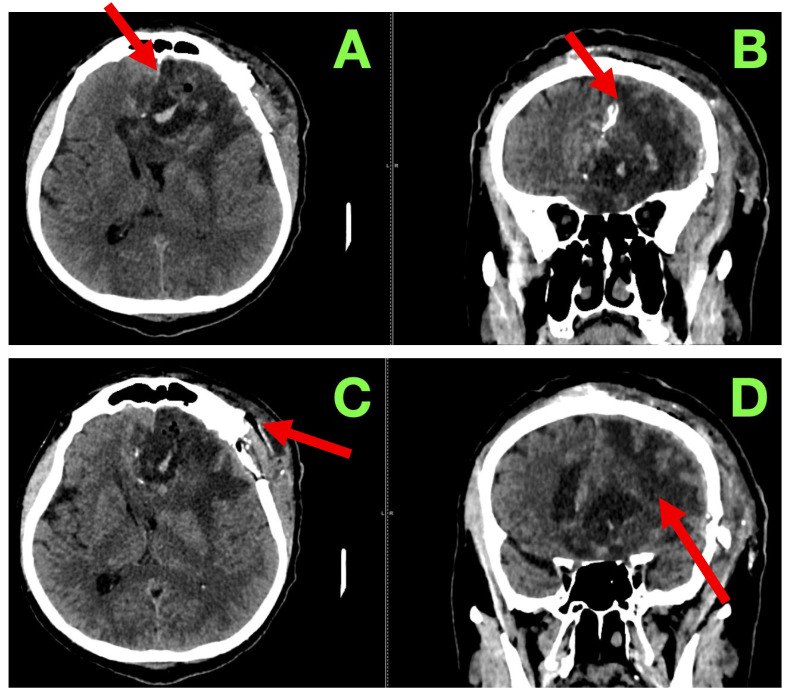
Immediate post-operative non-contrast CT scan. (**A**): Axial section showing a well-defined hypodense surgical cavity in the left frontobasal region (red arrow), corresponding to the zone of resection. No acute hemorrhage is present. A thin hyperdense rim along the cavity walls likely represents blood residue and oxidized cellulose (hemostatic material). (**B**): Coronal reconstruction demonstrating the vertical extent of the resection cavity (red arrow), which reaches the superior frontal gyrus and approaches—but does not breach—the genu of the corpus callosum. The midline remains centered, with no evidence of subfalcine or transtentorial herniation. (**C**): Lower axial slice illustrating the anterior margin of the resection cavity (red arrow). The basal ganglia and anterior limb of the internal capsule are preserved, and no new infarct or hematoma is detected. (**D**): Coronal view confirming decompression of the previously compressed left frontal horn (red arrow). No hydrocephalus or extra-axial collections are present. A subtle air–fluid level is visible within the cavity, consistent with expected early post-operative change.

A non-contrast cranial CT scan was obtained within 4 h of the procedure to evaluate the resection cavity and rule out acute complications (Figure 2).

The patient followed a predictable and favorable clinical course through the expected first 24 h of recovery following the procedure. Hemodynamically, all parameters were physiological: blood pressure was normotensive (MAP 85–95 mmHg), heart rate was regular (72–76 bpm), he was spontaneously breathing (unlabored at 16–18/min), oxygen saturation remained above 97% on room air, and he was afebrile. Additionally, the patient had a GCS of 15, exhibited an intact attention span, and was fully oriented in serial neurological examinations. He had no motor asymmetry, cranial nerve palsies, and no new focal deficits. There was no change in the phasic characteristics of previously observed non-fluent expressive aphasia (decreased verbal output with intact comprehension, repetition, and naming), and there was no disinhibition, irritability, or apathy. Pupils were equal and direct-light reactive bilaterally. Fundoscopy on post-operative day 1 revealed no papilledema, and there were no new visual complaints reported by the patient. A small volume of serosanguinous fluid (<20 mL) was drained from the subgaleal drain over the 24 h period. Given that there was no evidence of CSF leak, subgaleal tension or wound dehiscence, the drain was safely removed on post-operative day 2. The dressing was dry and intact, with normal apposition of the wound, and no evidence of dehiscence, infection, hematoma, or subcutaneous emphysema. Oral intake was resumed within the first 24 h, with normal swallowing and restored appetite. The patient was continent and did not experience urinary retention or bowel dysfunction. The patient was autonomously able to transition from standing to sitting in bed with assistance as needed without any orthostatic symptoms when verticalized. Laboratory values collected on day 1 post-operatively demonstrated a CRP value of 13.2 mg/L and a D-dimer value of 765 ng/mL, which were expected and within normal parameters for the early post-operative time frame. The patient’s hemoglobin value remained stable at 12.4 g/dL. The patient’s platelets, INR, aPTT, and electrolytes were normal. The patient’s liver and renal function results were within normal limits. The patient did not require any antipyretic or antimicrobial drugs. Thromboprophylaxis was started with enoxaparin 0.4 mL/day on post-operative day 1 and continued without complication. The patient remained on therapeutic anticonvulsant prophylaxis with 1000 mg/day of levetiracetam without any sedation or adverse outcomes relating to behavior. Dexamethasone was also continued at 8 mg/day and was tapered over the next ten days. The patient did not experience agitation, hyperglycemia or gastrointestinal intolerance that might have resulted in discontinuing treatment. The patient ambulated in the corridor with minimal assistance on post-operative day 3. The patient was engaged in supervised neurorehabilitation exercises to try and initiate language and executive function tasks. The patient showed good affective tone with no indications of frontal lobe dysfunction or apathy. His speech remained limited and spontaneous but purposeful. The patient was compliant and showed appreciation for his clinical course. He continued to ask questions and was fully aware of his prognosis. The patient was monitored overnight and did not experience any seizures, confusion or agitated periods. No anti-emetics were given. There were no signs of hyponatremia, and the fluid intake/output was normal. On day 5 after surgery the patient had returned to all self-care with minimal supervision. Upon examination, the surgical incision was dry, well-apposed, and was free of erythema, swelling or discharge. The staples were left intact, and no new dressing was to be applied. There was no CSF leak, subgaleal collection or delayed hemorrhage. While the clinical team anticipated a contrast-based MRI to rule out the possibility of new lesions, the team was delayed in scheduling the MRI. The clinical team ultimately decided to wait until a 3-month post-operative scan given stability neurologically, the initial clean scan, and lack of complications. On post-operative day 7 the patient was discharged home in stable condition, fully oriented cognitively, could mobilize independently, and had no new focal deficits. Upon discharge, the patient exhibited the same aphasia as prior with some level of comprehension intact and was able to produce a good deal of phrases at the same level. The patient was at full motor strength and coordination on the right (unilateral). There was no spasticity, clonus, or evidence of a pyramid release. The medications upon discharge included levetiracetam 1000 mg/day, dexamethasone (taper to 0), pantoprazole 20 mg/day, prophylactic enoxaparin, and outpatient follow-ups scheduled with neurosurgery, neurology, and neuro-oncology.

At our 3-month follow-up the patient showed clinical stability and evidence of recovery. The family indicated that there had been gradual improvement in verbal fluency; particularly, they reported spontaneous talk during routine interactions with family support. The patient was able to produce a few grammatically correct sentences but was still producing at a slower rate relatively. There were also no new neurological deficits, and the patient reported no seizures, syncope, or natural headaches. The patient was fully self-sufficient with their care, had returned to ambling without an assistive device, and the patient was contributing in a limited capacity to household items and chores.

**Figure 3:**
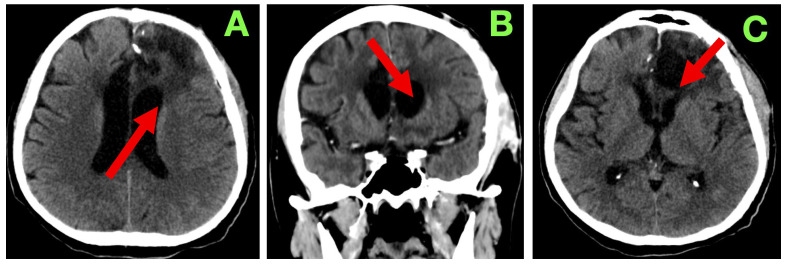
Three-month post-operative cranial CT scan. (**A**): Axial section demonstrating a well-defined left frontal resection cavity (red arrow) with smooth margins and no residual mass effect. Compared to the immediate post-operative stage, most of the surrounding vasogenic edema has resolved. Ventricular configuration is symmetric, with no midline shift or hydrocephalus. (**B**): Coronal reconstruction confirming stability of the resection cavity (red arrow), with no evidence of recurrent mass lesion. Cortical–subcortical architecture around the margins remains preserved, and no abnormal extracerebral collections are present. (**C**): Caudal axial slice showing the inferior aspect of the resection cavity (red arrow), with basal ganglia and thalamic anatomy intact. No delayed ischemia, hemorrhage, or secondary injury is observed, and the overall appearance is consistent with post-operative stability at three months.

On examination, the strength of motor function was intact bilaterally with no noticeable asymmetries or fasciculations or evident fatigability in the exam. The deep tendon reflexes were symmetric and there were flexor responses on the Planta response testing. Gait and coordination were normal on testing. On language examination, the patient showed fluent expressive output with mild anomia, but observed accuracy with respect to syntax, repetition, and reading comprehension. On cognitive assessment there was intact short-term memory, attention span, and executive function testing. As part of standard follow-up, a non-contrast cranial CT (Figure 3) was completed at 3 months. This scan showed a stable post-operative cavity without any new enhancement in the cavity or hemorrhagic complication or indication of any new hydrocephalus. There was also considerable reduction in perilesional edema. The ventricular appearance had returned to their apparent normal appearance, with no evident midline shift/sub-dural collection/sub-acute delayed infarct. Relative to the original report, they interpreted the findings as consistent with radiological stability of the surgical site and that there continued no progression, particularly with the absence of any new findings.

With a satisfactory clinical and radiological status on assessment, and with final molecular data pending, the case was put forward for multidisciplinary review to organize adjuvant oncological therapy. The purpose of this report is to illustrate a patient’s entire clinical, surgical, and immediate follow-up history with a large left frontal intra-axial tumor including the rationale for management, anatomical considerations, and the post-operative course as is typical in neurosurgical practice. A major emphasis will be on the degree of detail in microsurgical technique that is required to achieve maximal safe resection while retaining neurological function and quality of life. By describing the patient’s journey from presentation through to three-month follow-up demonstrating radiological stability, it is hoped that the importance of multidisciplinary care and coordination of medical professionals in achieving optimal outcomes and preparing for adjuvant therapy will be exemplified.

As of this report, three-month follow-up has been established and the patient remains alive, neurologically stable, and radiologically free of progression. The functional outcomes have been good: motor strength and cognition were indexed as preserved; language abilities continue to improve; and independence of activities of daily living have been maintained. The family have reported meaningful reintegration into every day and social life reflecting an acceptable quality of life.

After multidisciplinary discussion, the patient proceeded according to standard adjuvant therapy treatment and received adjuvant chemoradiotherapy according to the Stupp protocol, which included external beam radiotherapy in combination with concomitant and adjuvant temozolomide chemotherapy. The excellent neurological recovery following surgery allowed timely initiation of therapy with no further delay, which created continuity of care in a way that met internationally accepted standards of care. Transitioning from surgery to oncological treatment is made seamless when surgical methods of tumor resection depend upon knowledge of relevant anatomy as achieved through the use of microsurgical techniques and safe cytoreduction and can be completed while preserving functional state and eligibility for standard multimodal treatment of GBM.

## 3. Discussion

### 3.1. Anatomical and Functional Challenges in Dominant Frontal GBM Surgery

When resecting large frontobasal GBMs in the dominant hemisphere, the neurosurgeon is always weighing the maximal oncological clearance with the preservation of function. The surgical corridor will be dependent on not only tumor location, but the dynamic relationship the tumor has with cortical, subcortical, and vascular structures that govern higher-order cognitive processing and language processing [8]. The details of the cortical surface at the lesion may appear straightforward; yet, we know that eloquent cortices and white matter pathways are not only active, but they interact in complex ways as well. Connectomic studies have demonstrated that speech fluency, initiation, and semantics do not arise from a locus in the cortical area but are distributed across networks [9]. The inferior frontal gyrus (IFG) we now know consists of several functional subdivisions—pars opercularis and pars triangularis—that connect to other frontal and ventral areas via the frontal aslant tract (FAT) and the superior longitudinal fasciculus (SLF) [10]. These various pathways integrate with circuits that also include the basal ganglia and thalamus, which makes this area a risky part of the brain for deep dissection of a tumor. It was not our intent to perform intraoperative functional mapping; our approach consisted of incremental debulking while referencing stable anatomical landmarks, while continuously maintaining orientation from venous structures, the gyral landmarks, and the sulcal landmarks [11].

The frontobasal location presented additional challenges. The lesion was adjacent to the olfactory tract, orbitofrontal cortex, and the medial frontal gyrus—three regions of the brain associated with social behavior, personality, and executive function. Therefore, we performed our subpial dissection here to avoid these areas. The proximity to the anterior cerebral artery (ACA) and its perforating branches necessitated careful separation of the tumor from the falx and interhemispheric fissure while preserving blood supply. Injury to the microvasculature in this region could have impacted the cingulate cortex or supplementary motor area and resulted in akinetic mutism or a disabling supplementary motor area syndrome [12].

### 3.2. Microsurgical Techniques Performed Without Advanced Adjuncts

Although many advocate the use of neuronavigation, intraoperative MRI, and fluorescence-guided surgery (5-ALA) in countries with abundant resources, they each have limitations. For example, brain shift may decrease the accuracy of navigation. The 5-ALA technique has the advantage of identifying dense tumor zones but cannot identify diffuse infiltrating cells adjacent to healthy brain tissue. Awake mapping can provide insightful functional data, but this technique presents a logistical challenge and is not appropriate for every patient [13]. Our practice did not use adjuncts, not because we chose not to use them, but due to the reality of surgical practice in much of the world. We relied on the safety of an advanced knowledge of microsurgical anatomy developed through exposure to and practice with cadaveric and operative variants. The resection began with the non-eloquent cortex and advanced centripetally into the tumor core, while respecting the subpial and arachnoid planes around the deep vessels and tracts. This technique allowed us to spare function and minimize blood loss while maximizing a clear view.

Recent prospective studies have compared anatomy-only resections in eloquent gliomas with and without adjuncts. In the studies, authors reported that rates of gross total resection were not statistically different and that post-operative language outcomes were not worse when surgery was performed solely on the basis of anatomical topography [14]. These studies reiterate that surgical ability with respect to anatomy remains the main effect on successful outcomes. These studies have direct implications for surgeons who must adapt surgical strategy to the anatomy of the operation when advanced adjuncts are not available. To complement the present case with the evidence in the literature, we surveyed recent studies on resections of dominant frontal GBM with and without the use of adjuncts [15,16]. The comparative data below (Table 1) intends to highlight the variability in technique, functional preservation, molecular characterization, and survival outcomes, illustrating that in experienced hands, anatomy-driven microsurgery can achieve results comparable to adjunct-assisted strategies while preserving patient function.

### 3.3. Molecular Complexity and Reasons for Maximizing Cytoreduction

GBM, IDH-wildtype, is defined molecularly by any number of its hallmark changes, particularly a TERT promoter mutation, EGFR amplification (sometimes EGFRvIII), and the +7/−10 chromosome signature. In practice, the molecular reality is much more complex. Based on single-cell RNA sequencing from 2024 to 2025 datasets, a single tumor can contain different populations, i.e., mesenchymal, classical, proneural, etc, at the same time. The multi-phenotype plasticity allows subpopulations to develop escape to therapy, for a phenotype to change under treatment duress, and for the tumor bed to repopulate [29]. The mesenchymal phenotype (usually enriched at the invasive margin) is particularly resistant to radiotherapy via activation of downstream pathways, NF-κB and STAT3; conversely, the proneural can start with more treatment susceptibility. The proneural phenotype has been exhibited to transform to a more mesenchymal phenotype post treatment [30].

In addition to these molecular complexities, there are a number of prognostic markers emerging as key components in the clinical decision-making for GBM. IDH mutation status and MGMT promoter methylation still remain the best predictors of survival and response to temozolomide, respectively [31]. More recently, TERT promoter mutations and EGFR amplification, especially with chromosome +7/−10 changes, are other features that define the aggressive biology of IDH-wildtype tumors, while also serving as potential prognostic markers [32]. Age, performance status, functional outcomes post-operatively, and clinical variables remain important considerations for prognosis, eligibility for treatment considering their additive predictive variables, and also importantly, the recently proposed methylation-based tumor classification systems which are also all important stratifiers of risk and prognostic information, which allows better understanding of patient journeys at different time-points [33]. Collectively, it is clear that these molecular features and clinical variables highlight why the goal of surgery is maximal safe resection and how maximizing any potential subsequent adjuvant therapy will be improved [34].

So, while max safe resection is not just a cytoreduction, it is a molecular intervention. Beyond the surgical ‘unloading’ of the cellular ‘bucket’ during surgery, it would also unload those clones which may have resistance, which would only slow down the time to the eventual treatment-refractory recurrence. Additionally, cytokine signaling can be manipulated, and in fact especially in GBM [34]. The GBM tumor microenvironment is largely defined by a personalized immune evasion mechanism: M2-polarized, immunosuppressive tumor associated macrophages; and hypoxic perinecrotic niches with stem cell-like populations of aberrant blood vessels. With the increase in availability and increasing use of therapeutics, and other practices, the resection of the bulk tumor influences the resulting cytokine gradients (most importantly, positively impacting the efficacy of adjuvant immunotherapy strategies in these patients) [35].

Therapeutic pathways and functional preservation: With respect to patients’ functional status post-operatively, function is unsurprisingly the major determinant, and an aspect that can be considered pre-operatively, intraoperatively (when deciding what to leave behind, if anything), and most importantly, if post-operative complications arise in relation to reciprocal considerations for eligibility for adjuvant treatment eligibility. As a general rule, a 70+ KPS is needed to even go through most trials AND tolerate the Stupp regimen—max safe surgical resection, adjuvant focused radiation, then concurrent radiation + adjuvant temozolomide [36].

By the time an innovative, research-ready trial could be opened to recruit the patient, he had full motor function and intact language—stable cognition which permitted the adjuvant treatment to start at the appropriate timing [37].

Large cohorts and studies of management have all determined that the time to commencement of radiotherapy following surgery for patients with GBM is an independent predictor of patient survival, and initiation beyond six weeks from surgery is generally regarded to have poor outcome [38].

A relatively new clinical management pathway is also emerging which includes tumor treating fields—although a worse survival benefit (although compliance rate was better than averaging 18 h a day) was seen, along with convection-enhanced delivery (CED) for direct intraparenchymal chemotherapy and/or immunotherapy, personalized mRNA neoantigen vaccines that induce strong patient specific immune responses, and oncolytic virotherapy, which has a unique ability to potentially selectively lyse tumor cells, while also providing local immune stimulation from local immune cells [39,40]. Precision based radiotherapy—flexibly designed proton therapy, especially when delivering therapy with PET/MRI fusion guidance to allow hypofractionated regimes while better sparing the eloquent cortex—was also seen. All of these treatment pathways have all taken the one common prerequisite very seriously—function following resection—and they all recognize that microsurgical decision-making will create sequelae that will impact far beyond the operating room [41]. Imaging principles and monitoring for disease after surgery: In a clinical setting, immediate post-operative imaging is performed to diagnose potential surgical complications, while follow-up imaging is performed to find any early recurrences vs. post treatment abnormalities. In this case there was a CT performed at the week mark and then three monthly MRI-equivalent CTs to continue monitoring. In the 2024 meta-analyses, there is sufficient evidence to endorse CT over MRI in neurologically stable patients when there is reason to defer MRI until the first adjuvant therapy assessment for clinical and economic reasons.

In the context of interpreting follow-up imaging in GBM patients, medical professionals must be aware of pseudoprogression, where treatment-related enhancement of symptoms and recurrence can occur on MRI within the first three months of chemoradiation treatment. There were no new neurological signs, and cortical complication and otherwise stable or improved or recovering imaging is also what happened here, so the additional evidence was evidence as to why maintaining standard therapy would be appropriate without deviating from therapy.

### 3.4. Global Neurosurgical Context and Future Directions

Greater than 95% of GBM resections cannot or have depended on when well-described operational adjuncts to our surgical intent are without the emphasis from much of the neuro-oncology literature to rely on adjuncts that come from technology, and, as was the case here, the availability of surgical adjuncts is that they can only be considered in a consultative setting [42]. The broader implication of this case is that it illustrates how anatomy-driven microsurgery can remain a feasible and educationally valuable approach, particularly when advanced intraoperative adjuncts are unavailable. By emphasizing meticulous respect for cortical, subcortical, and vascular structures, such techniques may offer meaningful oncological and functional outcomes in carefully selected circumstances, while underscoring the continued relevance of fundamental microsurgical principles. The 2021 consensus from the World Federation of Neurosurgical Societies (WFNS) indicated that the importance of anatomical skills for a clinician was still a standard regardless of technology and provided a universal safety net for intraoperative technology failure, not to mention unwanted effects. There are unique identifiable competencies in having anatomical capital when developing practitioner-level surgical skill-sets which yield equitable surgical outcomes no matter the region or world we practice in [43].

The surgical environment will adopt a bulky replacement model of potential future adjuncts or, as an emerging concept, low-cost adjuncts such as low-cost portable high-frequency intraoperative ultrasound with AI-assisted segmentation, augmented reality overlays of DICOM from pre-operative imaging, or rapid intraoperative methylation profiling to classify tumor types in real-time [44]. Any combination and/or, more importantly, a combination of more low-cost adjuncts will only be useful when they are also nested in the new paradigm of anatomy-driven surgery and will lay the foundation for any surgical specialty to have standardized outcomes worldwide, especially for patients that should not merely be limited to the geography and distance to have the best access to safe maximal resections while considering functional objective outcomes [45].

## 4. Conclusions

This report discusses the management of a large, dominant frontal GBM that was resected using microscope alone with no intraoperative navigation, mapping, or adjunct fluorescence. This case study is an example of how a large extent of resection can be completed while also preserving relevant neurologic function through a careful pre-operative assessment, respect for cortical and subcortical anatomy, and disciplined micro-surgical technique.

Our experience does not seek to diminish the importance of the intraoperative adjuncts that have changed glioma surgery in many centers, but rather suggest that their absence is not a barrier to safe and successful outcomes when performing adequate attention to anatomical detail. In many areas of the world, where advanced techniques may not be available, it is vital for a neurosurgeon to adopt and move forward with a decision-making pathway that is driven by anatomy, rather than dismissing the use of anatomy as lacking merit and importance.

The preservation of motor and cognitive function, as well as the ability to communicate, allowed the patient to initiate adjuvant therapy in a timely manner, which is thought to have a significant impact on survival in GBM. The case presented here reinforces the notion that functional protection is a necessary accompaniment to oncological success.

We offer this case in the hope that it contributes to a common experience across the globe, shared internationally by neurosurgeons, and reinforces the process of refining methods—particularly in our evolving, technological medical environment—whether link-mounted, anatomically dictated, or both, with the ultimate intent to value a patient’s function and quality of life, above all else.

## Figures and Tables

**Table 1:** Summary of recent studies comparing surgical strategies for dominant frontal glioblastoma resection, detailing extent of resection, functional preservation, molecular profiles, and survival outcomes, with relevance to the present anatomy-driven, microscope-only case.

Strategy	Typical EOR	Functional Risk	Adjuvant Therapy Impact	Key Limitations	Resources	Relevance	References
Anatomy-only microsurgery	~90–95% (selected)	8–15% if deep tracts; ≤7% disciplined	8–15% language sequelae if deep tracts breached; ≤7% in disciplined series	No mapping; steep learning curve	Low-mod equip; high anatomy skill	Present case: high EOR, preserved function, timely CRT	[17,18]
Awake mapping	95–98%	3–7% (expert centers)	Highly favorable; early CRT	Patient tolerance; long OR; team logistics	High; specialized team	Alternative pathway; similar goals	[19,20]
Nav + 5-ALA	~95% CE zone	6–10% if over-extended	Favorable if restraint	Brain shift; misses non-CE	Moderate–high	Outcomes comparable to our case	[21,22]
iMRI	Highest GTR	5–9%	More complete resection	Cost, workflow, suite required	Very high	Not available in our setting	[23]
Supramaximal (FLAIR)	Rarely safe dominant IFG	High if near FAT/SLF	Neutral if deficits delay CRT	Language morbidity	High expertise	Not indicated in this case	[24]
LITT	Limited cytoreduction	Low morbidity; edema risk	Neutral/salvage	Seizures; MR suite	High	Salvage at recurrence	[25,26]
Endoscopic-assisted	Margin aid	Similar to open	Favorable in select	Bleeding control; learning curve	Moderate	Useful if interhemispheric	[27]
ioUS	Improves margin confidence	Indirect	Favorable	Operator-dependent	Low-cost	Practical adjunct	[28]

## Data Availability

The raw data supporting the conclusions of this article will be made available by the authors on request.
